# Transcriptomic Profiling of GRA47 Deletion in *Toxoplasma gondii* Reveals Transcriptional Reprogramming of Stress Adaptation and Metabolic Compensation

**DOI:** 10.3390/vetsci13060523

**Published:** 2026-05-28

**Authors:** Xing Tian, Xin-Sheng Lu, Jing-Ya Duan, Jing Li, Chen-Ran Tian, Xing-Quan Zhu, Xiao-Nan Zheng

**Affiliations:** Shanxi Key Laboratory of Animal Disease Research, Prevention and Control, College of Veterinary Medicine, Shanxi Agricultural University, Jinzhong 030801, China; txing2001@163.com (X.T.); luxinsheng2024@126.com (X.-S.L.); 18539037220@163.com (J.-Y.D.); lijing1127x@163.com (J.L.); chenrantian2019@163.com (C.-R.T.)

**Keywords:** *Toxoplasma gondii*, GRA47, transcriptomics, DEGs, RT-qPCR

## Abstract

Dense granule protein 47 (GRA47) of *Toxoplasma gondii*, which localizes to the parasitophorous vacuole membrane (PVM) and the parasitophorous vacuole (PV), is critical for PVM integrity, nutrient permeability, and parasite virulence. However, the global transcriptional response to GRA47 loss remains unexplored. High-throughput RNA sequencing was performed to compare transcriptomic profiles of a GRA47 knockout strain (RHΔ*gra47*) and its parental wild-type RH strain. Differentially expressed genes (DEGs) were identified and analyzed by Gene Ontology (GO) and KEGG enrichment, with selected DEGs validated by reverse transcription quantitative real-time PCR (RT-qPCR). A total of 285 DEGs were identified, comprising 218 upregulated and 67 downregulated genes. Upregulated DEGs were enriched in autophagy, mitophagy, ribosome biogenesis, and GPI anchor biosynthesis pathways, along with nutrient starvation response terms, which may suggest potential roles for the activation of stress response and compensatory metabolic programs. Downregulated DEGs were enriched in host cell entry, rhoptry components, membrane-related terms, and the Hedgehog signaling pathway, a pattern that could suggest a possible association with resource allocation strategy. This first transcriptomic analysis of GRA47 loss in *T. gondii* identifies transcriptional changes that point to a possible resource allocation strategy prioritizing parasite survival over proliferation and offers a foundational resource for investigating compensatory mechanisms and developing novel intervention strategies. This study reports transcriptional changes only; further experiments are needed to confirm protein-level effects and biological function.

## Simple Summary

*Toxoplasma gondii* is a zoonotic parasite that causes serious disease in immunocompromised animals and people. It survives within host cells in a specialized compartment called the parasitophorous vacuole. A parasite protein named GRA47 helps maintain this compartment. In this study, we investigated how the parasite regulates global gene expression in response to GRA47 loss by high-throughput RNA sequencing of a GRA47 knockout strain (RHΔ*gra47*) and its parental wild-type RH strain. Differentially expressed genes (DEGs) were identified and analysed by Gene Ontology (GO) and KEGG enrichment, and selected DEGs were validated by reverse transcription quantitative real-time PCR (RT-qPCR). We identified 285 DEGs, comprising 218 upregulated and 67 downregulated genes. The upregulated DEGs were enriched in autophagy, mitophagy, ribosome biogenesis, and GPI anchor biosynthesis pathways, along with nutrient starvation response terms, suggesting a possible link to the activation of stress response and compensatory metabolic programs. The downregulated DEGs were enriched in host cell entry, rhoptry components, membrane-related terms, and the Hedgehog signaling pathway, a pattern that may reflect a resource allocation strategy. These findings suggest extensive transcriptional reprogramming centered on stress adaptation and metabolic compensation. Though providing a foundation for studying the compensatory mechanisms, these findings are at the transcript level and require protein and functional validation.

## 1. Introduction

*Toxoplasma gondii*, an obligate intracellular apicomplexan parasite, has a complex life cycle involving sexual and asexual replication and infects humans and nearly all warm-blooded animals worldwide [1,2]. Although primary infection in immunocompetent individuals typically transitions to asymptomatic chronic infection due to effective immune responses, the persistence of tissue cysts (especially in the brain and muscles) poses a latent risk [3,4]. In immunocompromised patients and congenitally infected fetuses, toxoplasmosis can lead to life-threatening encephalitis, hydrocephalus, retinochoroiditis, and severe neurological disorders [5]. Thus, elucidating the molecular mechanisms underlying *T. gondii* pathogenicity is of great public health importance.

The pathogenicity of *T. gondii* relies on its ability to invade host cells, establish and modify its intracellular niche, the parasitophorous vacuole (PV), and finely regulate host cell functions [6]. These processes are highly dependent on a large arsenal of effector proteins secreted from three specialized organelles (rhoptries, micronemes, dense granules) [3,7]. Among them, the dense granule protein (GRA) family is central to remodeling the parasitophorous vacuole membrane (PVM) and the PV space. Different GRAs form channels on the PVM, construct tubular networks, or interact with host organelles and signaling pathways, collectively creating a unique niche that facilitates nutrient uptake, immune evasion, and long-term survival [8]. GRA47 is an important member of the dense granule family and localizes to the PVM and PV. Previous studies have shown that deletion of GRA47 reduces PVM permeability to small molecules, impairs parasite growth, and dramatically attenuates parasite virulence in mice [9,10], indicating its critical role in nutrient acquisition. However, current research has mainly focused on its direct role in PV morphology and PVM permeability [11,12]. Whether GRA47 knockout elicits broader transcriptional changes within the parasite and how these changes contribute to the observed phenotypic defect remain to be explored. Given that the PVM serves as the critical interface between the parasite and its host, alterations in its permeability may trigger adaptive transcriptional programs in intravacuolar tachyzoites [13,14]. Systematically uncovering the transcriptomic reprogramming triggered by GRA47 loss is key to understanding its multifaceted functions.

Knockout of a single gene in *T. gondii* can lead to widespread alterations in the expression of numerous other genes. Deficiency of TgAP2XII-9 led to the significant downregulation of rhoptry proteins and rhoptry neck proteins, causing severe defects in the invasion and egress of tachyzoites [15]. Inactivation of GRA1 caused bradyzoite-stage specific genes to account for 24% of all the upregulated genes [16]. Deletion of TgFBXL2 led to significant upregulation of merozoite marker proteins such as GRA80 and MIC17c, suggesting a role for TgFBXL2 in regulating sexual-stage gene expression [17]. These widespread transcriptional changes induced by gene deletion not only reflect the direct cellular response to perturbation, but also uncover functional associations among genes or suggest their involvement in common regulatory networks. Therefore, systematically dissecting these transcriptional alterations and the enriched signaling pathways represents a critical entry point for understanding the biological function of a given gene.

However, how GRA47 deletion systematically affects the global transcriptomic landscape of *T. gondii* and the underlying regulatory networks remains largely unexplored. In this context, our study aimed to systematically delineate the transcriptional reprogramming triggered by GRA47 loss and identify the biological processes and signaling pathways involved. To this end, we employed high-throughput RNA sequencing (RNA-seq) to compare the transcriptomic profile of a previously constructed GRA47 knockout strain (RHΔ*gra47*) with that of the wild-type RH strain, combined with bioinformatic functional enrichment and pathway analyses of differentially expressed genes. Our findings not only provide novel molecular insights into GRA47-mediated pathogenesis but also establish a theoretical foundation for the identification of potential intervention targets.

## 2. Materials and Methods

### 2.1. Parasite and Cell Line

The *T. gondii* RHΔ*ku80* strain (referred to as RH, the parental strain), the knockout strain RHΔ*ku80*Δ*gra47* (referred to as RHΔ*gra47*), and human foreskin fibroblasts (HFF) were maintained in the Shanxi Key Laboratory of Animal Disease Research, Prevention and Control, College of Veterinary Medicine, Shanxi Agricultural University. HFF cells were cultured in DMEM (Gibco, Suzhou, China) supplemented with 10% fetal bovine serum (FBS, Gibco, Thornton, NSW, Australia), 100 μg/mL streptomycin (Solarbio, Beijing, China), 100 U/mL penicillin (Solarbio, Beijing, China), and 10 mM HEPES (Solarbio, Beijing, China) at 37 °C under 5% CO_2_ [9,18].

### 2.2. Transcriptome Sequencing and Bioinformatics Analysis

Tachyzoites of the RH wild-type and RHΔ*gra47* strains were propagated in HFF cells in DMEM containing 2% FBS. When abundant plaques formed but parasites had not fully egressed, cells were scraped and collected. Three independent biological replicates were prepared for each strain. Total RNA was extracted using TRIzol reagent (Thermo Fisher Scientific, Waltham, MA, USA), and residual genomic DNA was eliminated using RNase-free DNase (Thermo Fisher Scientific, Waltham, MA, USA). RNA concentration and purity were assessed using an Agilent 2100 Bioanalyzer (Agilent Technologies, Santa Clara, CA, USA) and a NanoDrop spectrophotometer (Thermo Fisher Scientific, Waltham, MA, USA). The RNA-seq library was constructed following the manufacturer’s standard protocol, which involved mRNA isolation, fragmentation, cDNA synthesis, end repair, A-tailing, adapter ligation, PCR amplification, and purification with AMPure XP beads (Beckman Coulter, Brea, CA, USA). All libraries were constructed and sequenced in a single batch to minimize batch effects. Following sequencing on the BGI-AEQ platform (Shenzhen, China), raw reads were filtered to remove low-quality reads and adapters, and quality parameters such as Q30 values were assessed. The resulting clean reads were then aligned to the *T. gondii* ME49 reference genome (ToxoDB release 68; https://toxodb.org) using HISAT2 (v2.2.1) [19], and mapping rates were determined. Since host RNA was not depleted prior to sequencing, only reads that uniquely aligned to the *T. gondii* genome were retained for further analysis. Differential expression analysis was conducted using the DESeq2 package (v1.40.2) with an adjusted *p*-value (*Q*-value) ≤ 0.05 and |log_2_ fold change| ≥ 1 being considered differentially expressed [20,21]. Gene Ontology (GO) enrichment and Kyoto Encyclopedia of Genes and Genomes (KEGG) pathway analyses were conducted using the phyper function in R (v4.4.3) with a significance threshold of *p*-value ≤ 0.05. Principal component analysis (PCA) was performed on normalized count data in R to evaluate sample clustering and reproducibility.

### 2.3. Validation of RNA-Seq Data by Reverse Transcription Quantitative Real-Time PCR (RT-qPCR)

To verify the RNA-seq results, 16 key DEGs (8 upregulated and 8 downregulated) were randomly selected for RT-qPCR (Table S2). Total RNA (1 μg) was reverse transcribed using HiScript III RT SuperMix for qPCR (Vazyme, Nanjing, China). Specific primers were designed based on target gene sequences, and *T. gondii* β-tubulin gene was used as the endogenous reference for normalization. Three independent biological replicates were performed for each strain, and each RT-qPCR reaction was run in triplicate as technical replicates. RT-qPCR was carried out on a QuantStudio 3 Real-Time PCR System (Applied Biosystems by Thermo Fisher Scientific, Waltham, MA, USA) using ChamQ Universal SYBR qPCR Master Mix (Vazyme, Nanjing, China). The cycling protocol was: 95 °C for 30 s, followed by 40 cycles of 95 °C for 10 s, 60 °C for 30 s, and 72 °C for 15 s; melting curve analysis from 72 °C to 95 °C ensured product specificity. Relative expression levels were estimated using the 2^−ΔΔCt^ method [22]. Statistical comparisons between the RHΔ*gra47* and wild-type RH strains were performed using Student’s *t*-test, with a significance threshold of *p* < 0.05. Error bars represent the standard deviation (SD) of three biological replicates.

## 3. Results

### 3.1. Quality Control of RNA-Seq Data

**Figure 1:**
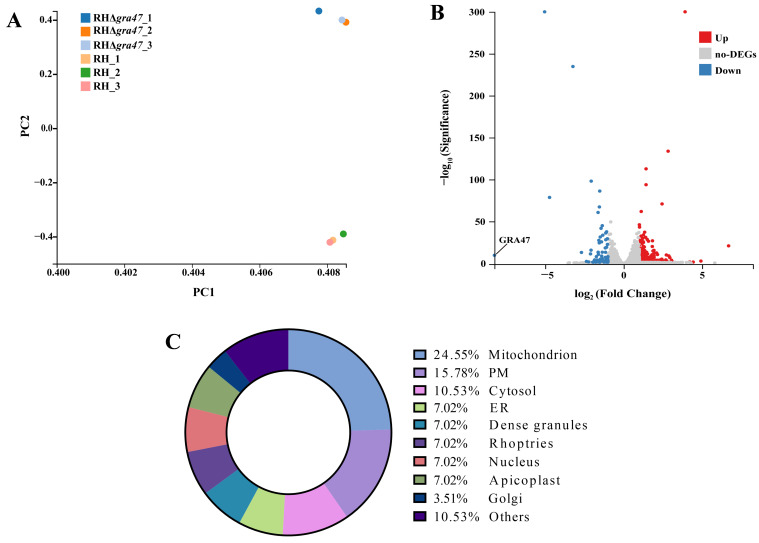
Transcriptomic overview of the RHΔ*gra47* strain and identification of differentially expressed genes (DEGs). (**A**) Principal component analysis (PCA) of RNA-seq samples. PCA was performed based on the expression levels of all genes. Each dot represents an individual biological replicate (n = 3 per group). Samples from the RH and RHΔ*gra47* strains formed two distinct clusters. (**B**) Volcano plot showing the distribution of DEGs. The *x*-axis represents the log_2_-transformed fold change, and the *y*-axis represents the statistical significance (−log_10_ transformed *p*-value). Red, blue, and gray dots denote upregulated, downregulated, and non-significant genes, respectively. (**C**) Predicted subcellular localization of DEG-encoded proteins. Subcellular distributions were analyzed based on annotation information from the ToxoDB database.

To assess the quality and reproducibility of the transcriptomic data, RNA-seq was performed on three independent biological replicates per strain (RH and RHΔ*gra47*). Quality control metrics are summarized in Table S1. Briefly, after stringent filtering, an average of 22.10 million clean reads per sample were obtained, with a Q30 value exceeding 92.56% for all samples. The average mapping rate to the *T. gondii* ME49 reference genome ranged from 19.38% to 32.95% across samples, due to the expected host (HFF) read contamination inherent to intracellular parasite transcriptomics. PCA revealed clear separation between RH and RHΔ*gra47* samples along the second principal component (PC2), with biological replicates of the same genotype clustering together (Figure 1A).

### 3.2. Identification of DEGs

Differential expression analysis between RHΔ*gra47* and wild-type RH identified 285 DEGs (*Q* ≤ 0.05, |log_2_ FC| ≥ 1), consisting of 218 upregulated (76.5%) and 67 downregulated (23.5%) genes. As shown in the volcano plot (Figure 1B), most DEGs exhibited moderate fold changes (|log_2_ FC| between 1 and 3), with a clear bias toward upregulation. The full list of DEGs is provided in Table S3. To explore the potential functional implications from a subcellular perspective, we analyzed the predicted localization of proteins encoded by DEGs based on the hyperLOPIT data from ToxoDB database [23]. The largest fraction of DEG products were predicted to localize to mitochondria (24.55%), followed by plasma membrane (15.78%), cytosol (10.53%), and endoplasmic reticulum (7.02%), dense granules (7.02%), rhoptries (7.02%), nucleus (7.02%), apicoplast (7.02%), Golgi (3.51%), and other compartments (10.53%) (Figure 1C).

### 3.3. RT-qPCR Validation of DEGs

**Figure 2:**
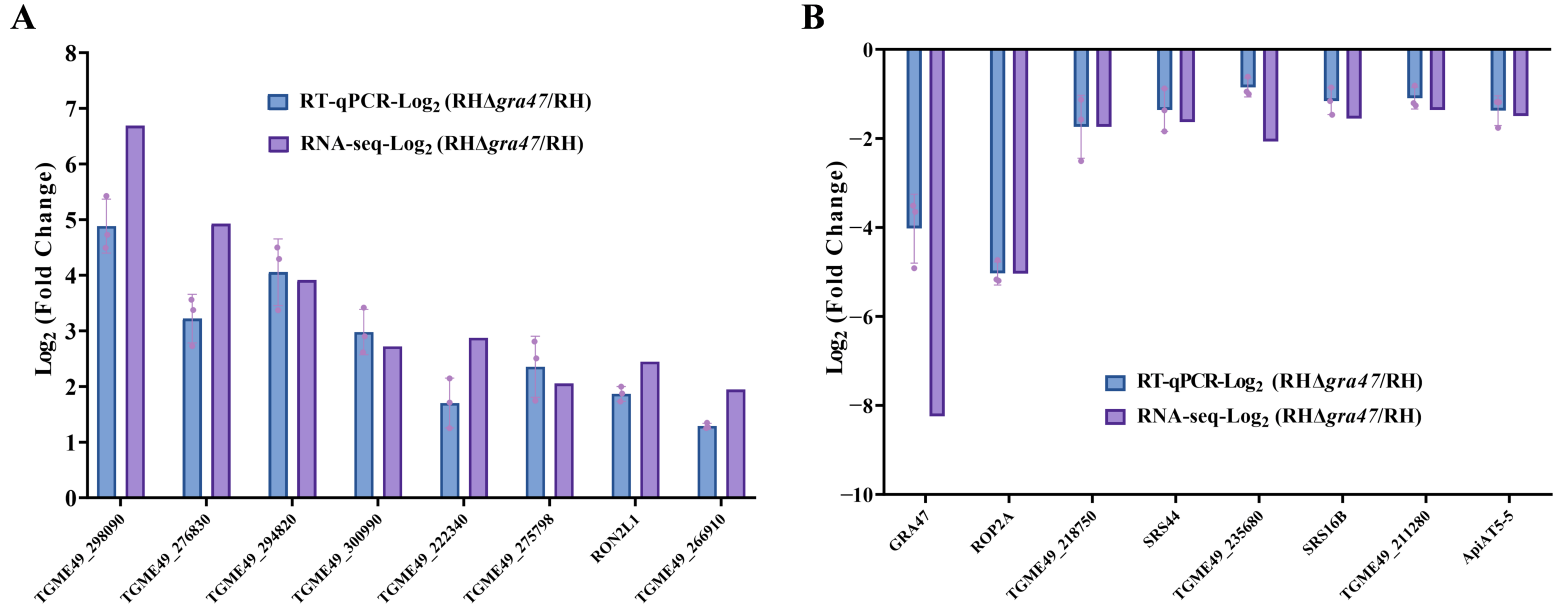
Validation of 16 randomly selected differentially expressed genes (DEGs) in RHΔ*gra47* by RT-qPCR. (**A**) Expression analysis of eight upregulated differentially expressed transcripts. (**B**) Expression analysis of eight downregulated differentially expressed transcripts.

To validate the RNA-seq data, 16 DEGs encoding various proteins, including GRAs, rhoptry proteins, and SAG-related sequences, were randomly selected for RT-qPCR analysis, with β-tubulin being used as an internal control. As shown in Figure 2A, eight genes (e.g., *TGME49_298090*, *TGME49_276830*, and RON2L1) exhibited significantly higher transcript levels compared to the wild-type RH strain. Conversely, GRA47, ROP2A, SRS44, ApiAT5-5, and four other genes showed reduced expression (Figure 2B). The expression patterns of all 16 genes were consistent with the RNA-seq data in terms of upregulation or downregulation, thereby confirming the reliability of the transcriptomic results.

### 3.4. GRA47 Knockout Results in Transcriptomic Upregulation of Stress Response and Cellular Processes Remodeling

**Figure 3:**
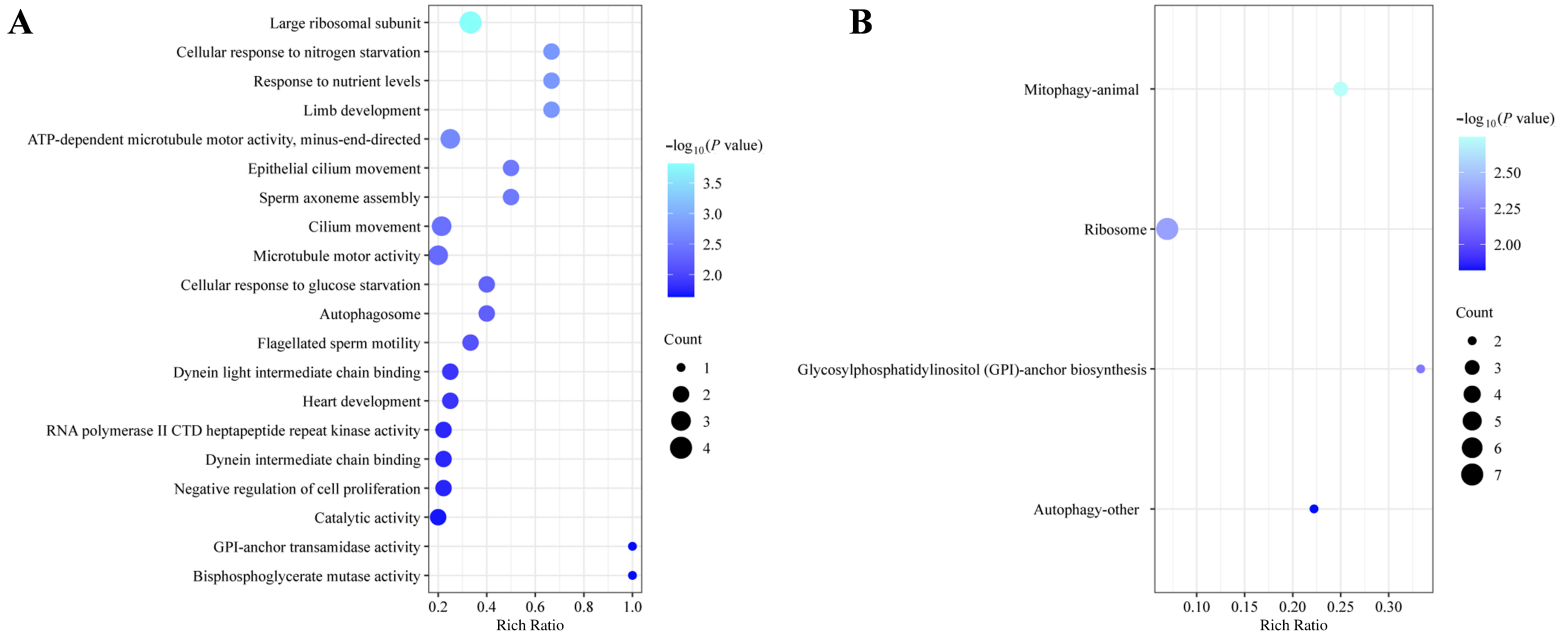
GO and KEGG enrichment analyses of upregulated differentially expressed genes. (**A**) Top 20 GO terms ranked by significant (*p*-value). (**B**) Top 4 KEGG pathways ranked by significant (*p*-value).

To visualize the overall functional landscape of the 218 upregulated DEGs, GO and KEGG enrichment analyses were performed, with key results summarized in Figure 3A. GO enrichment analysis revealed significant enrichment in functions associated with protein synthesis and microtubule-driven motor activity. These enriched terms included: large ribosomal subunit (*p* = 1.53 × 10^−4^, count = 4), ATP-dependent microtubule motor activity (minus-end-directed) (*p* = 2.30 × 10^−3^, count = 3), cilium movement (*p* = 3.90 × 10^−3^, count = 3), and microtubule motor activity (*p* = 4.52 × 10^−3^, count = 3). Additionally, terms such as cellular response to nitrogen starvation (*p* = 1.66 × 10^−3^, count = 2), response to nutrient levels (*p* = 1.66 × 10^−3^, count = 2), and autophagosome (*p* = 5.85 × 10^−3^, count = 2) were also significantly enriched (Table S4).

KEGG pathway enrichment analysis further identified four significantly enriched pathways among the upregulated DEGs, as presented in Figure 3B and Table S5: mitophagy-animal (*p* = 1.80 × 10^−3^, count = 3), ribosome (*p* = 4.40 × 10^−3^, count = 7), GPI anchor biosynthesis (*p* = 6.57 × 10^−3^, count = 2), and autophagy-other (*p* = 1.51 × 10^−2^, count = 2). Among these, mitophagy displayed the most significant enrichment, consistent with the GO identified autophagosome term. The enrichment of GPI anchor biosynthesis aligns with the GO term GPI anchor transamidase activity.

Together, the absence of GRA47 led to the upregulation of 218 genes, many of which are associated with stress response and metabolic pathways.

### 3.5. Downregulated Genes Are Associated with Host Invasion, PVM Integrity, and Developmental Signaling Pathways

**Figure 4:**
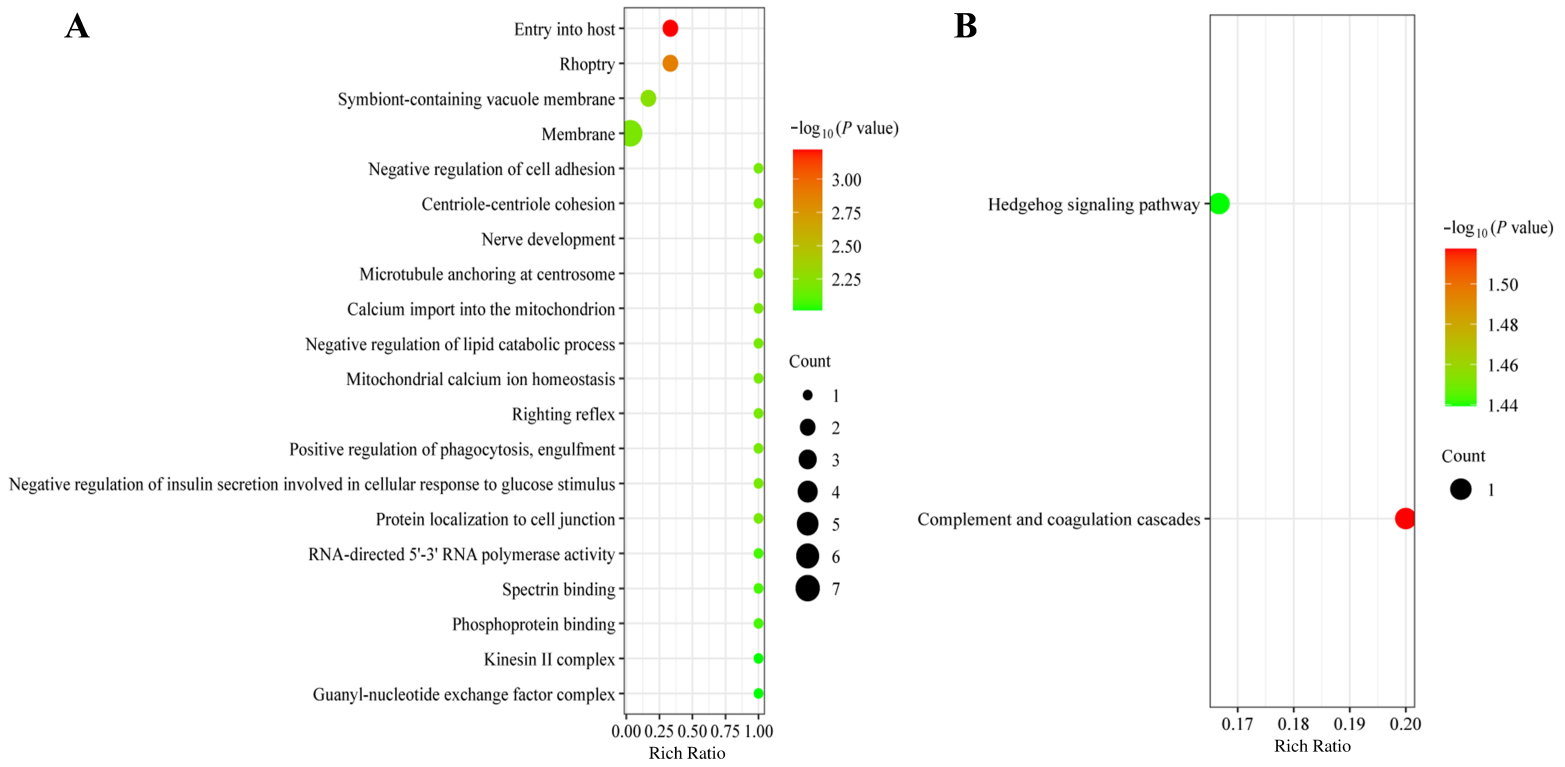
GO and KEGG enrichment analyses of downregulated differentially expressed genes. (**A**) Top 20 GO terms ranked by significant (*p*-value). (**B**) Top 2 KEGG pathways ranked by significant (*p*-value).

The 67 downregulated DEGs were subjected to the same enrichment analyses, with results illustrated in Figure 4A. Significant enrichment was observed in functional terms, including membrane (*p* = 6.30 × 10^−3^, count = 7), entry into host (*p* = 6.01 × 10^−4^, count = 2), symbiont-containing vacuole membrane (*p* = 5.67 × 10^−3^, count = 2), and rhoptry (*p* = 1.34 × 10^−3^, count = 2) (Figure 4A, Table S4). In addition, several other enriched terms were observed, each comprising a single gene, including negative regulation of cell adhesion, centriole-centriole cohesion, calcium import into the mitochondrion, and mitochondrial calcium ion homeostasis (all terms with *p* = 6.59 × 10^−3^, count = 1) (Table S4). Collectively, these results indicate that GRA47 deletion predominantly downregulates transcription levels of genes involved in host invasion and membrane-related processes, while the broader spectrum of enriched terms, though each comprising only a single gene, suggests that GRA47 loss may also exert pleiotropic effects on other cellular processes.

KEGG pathway enrichment analysis demonstrated that downregulated DEGs were significantly enriched in two pathways: the Hedgehog signaling pathway (*p* = 3.64 × 10^−2^, count = 1) and complement and coagulation cascades (each with *p* = 3.04 × 10^−2^, count = 1) (Figure 4B, Table S5). These findings expand our understanding of the transcriptional consequences downstream of GRA47 loss.

## 4. Discussion

GRA47 has recently been identified as a key effector localized to the PVM and PV of *T. gondii*, and its deletion leads to abnormal PV morphology, reduced PVM permeability to small molecules, and markedly attenuated virulence [9]. However, how the parasite globally modulates its gene expression in response to GRA47 loss remains unexplored. Here, we present the first transcriptomic analysis of the RHΔ*gra47* strain, revealing extensive transcriptional reprogramming and providing a new dimension to our understanding of GRA47 functions.

The identification of 285 DEGs strongly indicates that loss of GRA47 influences the parasite’s transcriptional program, likely through feedback mechanisms triggered by PVM dysfunction. Such autonomous transcriptional reprogramming may represent an adaptive survival strategy to cope with the altered intra-PV microenvironment, including nutrient scarcity, waste accumulation, and osmotic imbalance, resulting from GRA47 loss [24,25]. Importantly, enrichment results are preliminary and were not experimentally validated; they serve solely to generate hypotheses.

Impaired membrane permeability is thought to induce nutrient stress, which in turn triggers autophagic flux to recycle cellular components and compensate for the reduced uptake of host-derived nutrients [26,27]. Consistent with this framework, our transcriptomic profiling reveals the upregulation of genes enriched in autophagic and mitophagic pathways. While previous studies have shown that deletion of key PVM pore-forming proteins, such as GRA17 and GRA72, abrogates molecular permeability [11,28], direct experimental validation of this conclusion in our context is still required. Such validation could be achieved through approaches including autophagic flux assay, mitochondrial morphological analysis, and conditional knockout studies.

The upregulation of genes involved in response to nitrogen and glucose starvation is associated with transcriptional evidence of nutrient stress in RHΔ*gra47* parasites. Of note, despite this nutrient-limited condition, the parasites appear to attempt to counter the stress by upregulating ribosomal proteins (ribosome pathway), likely as a compensatory mechanism to enhance translational capacity [29]. However, this adaptive response appears insufficient to rescue the growth defects observed in RHΔ*gra47* in our previous study. This is presumably due to a sustained shortage of essential building blocks, such as amino acids or nucleotides, which may not be overcome by ribosomal upregulation alone.

The most striking downregulated DEGs were associated with membrane, rhoptry components, and entry into host. Rhoptries are specialized secretory organelles essential for host cell invasion and PV establishment [3,7]. In RHΔ*gra47*, genes encoding rhoptry components—notably ROP2A and ROP8—were among the most significantly downregulated DEGs. This association is linked to the near-complete loss of virulence observed in RHΔ*gra47* parasites. GRA47 itself resides on the PVM and PV, and its absence directly compromises PVM integrity [9,30]. Of note, the downregulation of invasion-related genes, which are typically required at the early stage of infection prior to PVM formation, is consistent with the possibility that the impact of GRA47 loss might extend beyond its immediate localization. One possible interpretation could be that the parasite might employ an adaptive resource allocation strategy in response to PVM dysfunction. Specifically, when GRA47 deletion impairs PVM permeability and limits nutrient acquisition, the parasite could potentially downregulate energetically costly invasion-related processes, such as the expression of rhoptry proteins and surface antigens (e.g., SRS family members), as a potential mechanism to prioritize energy and resources for survival under nutrient stress [31,32,33]. However, it is important to note that these considerations are primarily based on transcriptomic data and remain speculative at this stage. Direct experimental validation—for instance, through functional assays assessing invasion efficiency, rhoptry protein secretion, or targeted re-expression of key downregulated genes—would be necessary to conclusively establish a causal link between GRA47 deletion, transcriptional remodeling of invasion-related genes, and the observed virulence defect.

Beyond its impact on invasion-related genes, GRA47 deletion appears to affect genes involved in developmental signaling pathways. The downregulation of Hedgehog pathway components identified in our KEGG enrichment analysis is noted, though it should be interpreted with caution, as these annotations rely on sequence homology rather than experimentally verified functional conservation in *T. gondii*. If these genes do function in a Hedgehog-like capacity, their downregulation could be consistent with a possible link to abnormalities in cell cycle progression or differentiation [34]. This observation is particularly of interest in light of the observation that GRA47 deletion leads to a marked reduction in brain cyst burden in infected mice, a key indicator of chronic infection and bradyzoite differentiation [9]. Given that the Hedgehog pathway has been implicated in developmental transitions in other eukaryotes [35], it is plausible that its downregulation in RHΔ*gra47* parasites contributes to defective differentiation capacity, thereby providing a potential link between the transcriptomic changes observed in vitro and the attenuated chronic infection phenotype in vivo. Further investigation into the role of Hedgehog-related genes in *T. gondii* differentiation and cyst formation would be warranted.

Given the critical role of GRA47 in maintaining PVM permeability and the prominent enrichment of ribosome-related GO terms among upregulated genes, we prioritized five upregulated ribosomal protein genes RPL12, RPS10, RPS20, RPL17, and *TGME49_252270* for further consideration. Notably, these genes exhibit highly negative fitness scores in genome-wide CRISPR screens (ranging from −5.22 to −3.56), indicating their essentiality for parasite growth under standard culture conditions. Their transcriptional upregulation in the absence of GRA47 could represent a compensatory attempt to sustain protein synthesis capacity under nutrient-limited conditions [36], thereby offering a potential entry point to investigate how the parasite might cope with stress derived from a PVM permeability defect. However, we emphasize that the identification of negative fitness scores alone does not demonstrate their functional compensation in the GRA47-null background; these genes remain candidate targets whose specific roles in stress adaptation require direct validation through conditional knockout, overexpression, or chemical inhibition studies.

Similarly, given that global metabolic reprogramming would rely on coordinated regulation of enzyme expression and activity, we also identified upregulated enzyme encoding genes from significantly enriched KEGG pathways in the RHΔ*gra47* strain. These include genes involved in carbohydrate metabolism (PGM3 and PGMII) [37,38], as well as a putative GPI-anchor transamidase (*TGME49_252440*) possibly involved in glycan biosynthesis and metabolism [39], a putative proteasome subunit beta type-2 (*TGME49_254900*) possibly associated with amino acid metabolism and protein turnover, and a COX19 cytochrome oxidase assembly family protein (*TGME49_254260*) possibly linked to energy metabolism [40]. Like the ribosomal protein genes, these also carry negative CRISPR fitness scores (ranging from −5.07 to −0.97), underscoring their potential importance in maintaining parasite fitness under normal conditions. Their transcriptional level induction upon GRA47 deletion is consistent with the possibility that the parasite may activate specific metabolic and protein homeostasis pathways to compensate for impaired nutrient acquisition caused by PVM dysfunction. Of note, the upregulation of COX19, a gene involved in mitochondrial respiratory chain assembly, may reflect a compensatory effort to sustain mitochondrial function following the activation of mitophagy observed in our transcriptomic data [40]. Nonetheless, these candidate genes still require further direct experimental validation.

Collectively, these upregulated genes functionally linked to protein synthesis and core metabolism represent promising candidates for future mechanistic studies. Further investigation of these genes could provide insights into the compensatory strategies employed by *T. gondii* to survive under nutrient stress and may also help elucidate the downstream pathways through which GRA47 might support PVM function and parasite virulence.

In summary, this study provides a transcriptomic landscape of the adaptive response triggered by GRA47 deletion in *T. gondii*. Our data are consistent with the idea that GRA47 functions in maintaining PVM nutrient permeability rather than acting as a direct transcriptional regulator. Its loss appears to induce a state of nutrient stress, to which the parasite responds by orchestrating broad transcriptional reprogramming. The coordinated upregulation of stress response, autophagy, and metabolic compensation pathways, together with the downregulation of invasion-related genes, reveals a potential resource allocation strategy that may prioritize survival over proliferation. These findings not only illuminate the multifaceted roles of GRA47 in sustaining PVM function, but also suggest compensatory mechanisms that could support parasite fitness under adverse conditions.

Several limitations should be considered. First, differential transcript abundance does not necessarily reflect protein abundance or activity. We did not validate any findings at the protein-level. Second, our RT-qPCR validation confirms expression changes but does not assess functional consequences. Third, the enrichment analyses are exploratory and hypothesis-generating. Therefore, the conclusions regarding GRA47 function remain provisional until complemented by protein-level and phenotypic studies. Future work combining functional validation of candidate genes (e.g., conditional knockout or overexpression) with biochemical approaches (e.g., proximity labeling or metabolomics) will be essential to dissect the molecular pathways through which *T. gondii* senses and responds to PVM-derived stress [41,42,43]. Nevertheless, our findings establish a foundational framework for exploring parasite metabolic resilience and may inform the development of novel intervention strategies targeting compensatory pathways.

## 5. Conclusions

This study provides a comprehensive transcriptomic landscape of the adaptive response triggered by GRA47 deletion in *T. gondii*. We observed broad transcriptional reprogramming characterized by the upregulation of stress response, autophagy, and metabolic compensation pathways alongside the downregulation of energetically costly invasion-related genes, a pattern that could reflect a resource allocation strategy that may prioritize survival over proliferation. The DEGs identified, particularly those with negative CRISPR fitness scores, may serve as valuable candidates for future mechanistic investigations into how *T. gondii* senses and adapts to PVM-derived stress. Future work would need to move beyond transcript-level inference toward protein-level and functional validation to experimentally test the compensatory mechanisms proposed here, thereby contributing to the elucidation of GRA47-mediated pathogenesis and potentially informing the development of novel anti-toxoplasmosis interventions that target compensatory pathways.

## Data Availability

The data presented in this study are openly available in the NCBI Sequence Read Archive (SRA) under the accession number PRJNA1446183.

## Supplementary Materials

The following supporting information can be downloaded at: https://www.mdpi.com/article/10.3390/vetsci13060523/s1. Table S1: Summary of RNA-seq data quality for RH and RHΔ*gra47* samples; Table S2: The primers used in the RT-qPCR experiment; Table S3: Differentially expressed genes in RHΔ*gra47* strain (|Log2(RHΔ*gra47*/RH)| ≥ 1, Q values ≤ 0.05); Table S4: Differentially enriched GO terms in RH vs. RHΔgra47; Table S5: Differentially enriched KEGG pathways in RH vs. RHΔ*gra47*.
