# Supplementary material for: Transcriptomic Profiling of GRA47 Deletion in Toxoplasma gondii Reveals Transcriptional Reprogramming of Stress Adaptation and Metabolic Compensation
Source: Vet Sci. 2026 May 28;13(6):523. doi: 10.3390/vetsci13060523 (PMC13307739; doi:10.3390/vetsci13060523)
Supplement: Supplementary file 1 [file vetsci-13-00523-s001.zip › Table S1.pdf]

Table S1. Summary of RNA-seq data quality for RH and RHΔ*gra47* samples

Sample	Genotype	Replicate	Total Row Reads (Million)	Total Clean Reads (Million)	Clean Reads Ratio (%)	Total Clean Bases (Gb)	Clean Reads Q30 (%)	Mapping Rate (%)
RHΔ*gra47*_1	RHΔ*gra47*	1	23.2	22.11	95.30	6.63	93.16	19.38
RHΔ*gra47*_2	RHΔ*gra47*	2	23.2	22.12	95.34	6.64	93.16	23.84
RHΔ*gra47*_3	RHΔ*gra47*	3	23.2	22.14	95.43	6.64	92.98	32.95
RH_1	RH	1	22.88	22.14	96.77	6.64	93.13	32.50
RH_2	RH	2	22.88	22.06	96.42	6.62	92.56	32.26
RH_3	RH	3	22.72	22.03	96.96	6.61	92.61	25.86
