# Supplementary material for: Transcriptomic Profiling of GRA47 Deletion in Toxoplasma gondii Reveals Transcriptional Reprogramming of Stress Adaptation and Metabolic Compensation
Source: Vet Sci. 2026 May 28;13(6):523. doi: 10.3390/vetsci13060523 (PMC13307739; doi:10.3390/vetsci13060523)
Supplement: Supplementary file 1 [file vetsci-13-00523-s001.zip › Table S2.pdf]

Table S2. The primers used in the RT-qPCR experiment

Gene ID	Gene description	Forward primer (5′ -3′)	Reverse primer (5′ -3′)
TGME49_298090	Toxoplasma gondii family B protein	AGAAGGGAATGATGAAAGCGACAAG	ACACTTTCCTTTGCGGCTCAAC
TGME49_276830	hypothetical protein	GGCGTCACCGAGCGTAGAG	GGCGACTTCTCCAGCGTATTG
TGME49_294820	type I fatty acid synthase, putative	CATACCGCCTTGTTCGCCTTC	TGCCGCCACCAACTCACC
TGME49_300990	Toxoplasma gondii family C protein	GGAGAGGAGGGCACCACAGTC	GCCGTACAATTCATTCGCACCAC
TGME49_222340	NOL1/NOP2/sun family protein	TCAAGCACAGGCGGAGACAG	GCGTTGGCGGATGAACACAG
TGME49_275798	microneme protein, putative	TTCTTCAGACGCACTCGCACTTG	TGTACGCTTCTCGGCAAATCCAC
TGME49_294400	rhoptry neck protein RON2L1	CGCCGCCGAGACTGAGAC	CTGTGCCGCCCTTCTTTGTG
TGME49_266910	cell-cycle-associated protein kinase GSK, putative	TCCAGCCACACACTTTCGCATC	CCAGACACGGCATCAGTGACAAG
TGME49_254000	dense granule protein GRA47	TCGGTCGCCAACTTCCTTCC	CAGCACCACCCGTTTGATGTC
TGME49_215785	rhoptry protein ROP2A	TTAGAAGGTTACTCAGGCGTTTGC	AGGCGTCTGCGGTGGTTC
TGME49_218750	nucleoside triphosphate hydrolase, putative	AGTGGCTCAGCAATGGTTCAGTG	GAATCAGGGCGTGTGGCAAGG
TGME49_264660	SAG-related sequence SRS44	GCTCAATGTTCCTCGGCTTC	TCTACCATTCCTCCTCTGACCTTC
TGME49_235680	peptidase M16 inactive domain-containing protein	ACTTCCACCACACTGCCTACAC	CCTCGTTCTTCTCCTCCCTTAGC
TGME49_320190	SAG-related sequence SRS16B	TACCACTGCGGAACAGAAAG	ATGTACACCCGACGTCAAAG
TGME49_211280	hypothetical protein	ATCGCACGCCGCACTTTAGC	GCAGCCCTTTGAGACAGCCATC
TGME49_293420	apicomplexan amino acid transporter ApiAT5-5	GGCGTTCGTGCTGCTGTC	ATGATCTGGTTCGCTTCGTATGC
